# New fungal defensin-like peptides provide evidence for fold change of proteins in evolution

**DOI:** 10.1042/BSR20160438

**Published:** 2017-01-13

**Authors:** Yucheng Wu, Bin Gao, Shunyi Zhu

**Affiliations:** Group of Peptide Biology and Evolution, State Key Laboratory of Integrated Management of Pest Insects & Rodents, Institute of Zoology, Chinese Academy of Sciences, 1 Beichen West Road, Chaoyang District, Beijing 100101, China

**Keywords:** Gene discovery, cysteine-stabilized α-helical and β-sheet fold, structural evolution, exon-intron organization, phytopathogenic fungus

## Abstract

Defensins containing a consensus cystine framework, Cys_[1]_…Cys_[2]_X_3_Cys_[3]_…Cys_[4]_… Cys_[5]_X_1_Cys_[6]_ (X, any amino acid except Cys; …, variable residue numbers), are extensively distributed in a variety of multicellular organisms (plants, fungi and invertebrates) and essentially involved in immunity as microbicidal agents. This framework is a prerequisite for forming the cysteine-stabilized α-helix and β-sheet (CSαβ) fold, in which the two invariant motifs, Cys_[2]_X_3_Cys_[3]_/Cys_[5]_X_1_Cys_[6]_, are key determinants of fold formation. By using a computational genomics approach, we identified a large superfamily of fungal defensin-like peptides (fDLPs) in the phytopathogenic fungal genus – *Zymoseptoria*, which includes 132 structurally typical and 63 atypical members. These atypical fDLPs exhibit an altered cystine framework and accompanying fold change associated with their secondary structure elements and disulfide bridge patterns, as identified by protein structure modelling. Despite this, they definitely are homologous with the typical fDLPs in view of their precise gene structure conservation and identical precursor organization. Sequence and structural analyses combined with functional data suggest that most of *Zymoseptoria* fDLPs might have lost their antimicrobial activity. The present study provides a clear example of fold change in the evolution of proteins and is valuable in establishing remote homology among peptide superfamily members with different folds.

## Introduction

Defensins are small cysteine-rich, multifunctional peptides that are extensively distributed in multicellular organisms, including plants, animals and microorganisms. They mostly exert functions in host defence as antimicrobial and/or immune regulatory components although some other activities are also reported, e.g. protease inhibition and zinc tolerance [1]. These molecules exhibit enormous sequence and structural diversity with at least two distinct protein superfamilies, named *cis*-and *trans*-defensins, based on the orientation of the most conserved pair of disulfide bridges [2]. The *cis*-defensins refer to a group of structurally conserved peptides isolated from plants, fungi and invertebrates whereas the *trans*-defensins contain structurally diversified members, including big defensins from invertebrates and α-, β- and θ-defensins from vertebrates [2,3]. As one of the most diverse organisms on earth, fungi are emerging as a new source for exploring bioactive compounds, such as defensins [4]. Plectasin, micasin and eurocin are three representative fungal defensin-like peptides (fDLPs) that have been thoroughly studied in terms of their structures, functions and therapeutic potential [5–7]. Bearing the canonical defensin cystine framework “Cys_[1]_…Cys_[2]_X_3_Cys_[3]_…Cys_[4]_…Cys_[5]_X_1_Cys_[6]_” (X, any amino acid; …, variable residue numbers), they adopt a so-called cysteine-stabilized α-helix and β-sheet (CSαβ) fold, in which the Cys_[2]_X_3_Cys_[3]_ motif spans the α-helix and is connected to the C-terminal β-strand covering Cys_[5]_X_1_Cys_[6]_ via two disulfide bridges (Cys_[2]_–Cys_[5]_/Cys_[3]_–Cys_[6]_) and the third disulfide bridge (Cys_[1]_–Cys_[4]_) links the N-terminus to the first β-strand (cysteines numbered according to micasin) [7]. These three fDLPs belong to the ancient invertebrate-type defensins (AITDs) with activity on some antibiotic-resistant bacteria. Recently, it was found that some fDLPs classified into the classical insect-type defensins (CITDs) had gained ability in regulating fungal growth accompanying the loss of antibacterial function [8,9]. These classical fDLPs might be originated from gene duplication of the evolutionarily more ancient AITDs followed by functional diversification. Such ‘neofunctionalization’ is also observed in some plant and invertebrate defensins with versatile biological activities [10]. For example, the defensin scaffold in scorpion has evolved into ion channel-targeted neurotoxins for predation and defence [11].

In addition to AITDs and CITDs, our previous studies conducted by a computational genomics method identified six other families of fDLPs with unknown biological functions [7,12,13]. With the accumulation of a wealth of fungal genomes, data mining to find more new fDLPs on a genome-wide scale has become possible, which will help uncover the structural and functional diversity of fDLPs and shed new light on the evolutionary relationship among different defensin families. In this work, we carried out genomic database searching of a newly proposed fungal genus named *Zymoseptoria* and identified a total of 195 fDLPs with low sequence similarity to other peptides characterized so far except six cysteines. A combination of sequence and structural analyses leads to the discovery of structural divergence of fDLPs at the fold level. To the best of our knowledge, this is the first report on fold change of defensins in evolution.

## Materials and methods

### Database searches

The search strategies used here have been described previously [12]. Briefly, some representatives of known defensins from diverse organisms were used as queries to perform the TBLASTN search of the fungal genome sequences (http://www.ncbi.nlm.nih.gov/) under default parameters. New hits were also taken as queries until no hits appeared. To ensure secretion, retrieved sequences were filtered to screen members containing an N-terminal signal peptide (http://www.cbs.dtu.dk/services/SignalP/).

### Transcriptional analyses

To valid the transcriptional activity and the exon–intron boundary of the predicted defensin genes, SRA-Blast in NCBI (http://www.ncbi.nlm.nih.gov/) was performed against the released RNA-seq databases.

### *Ab initio* 3D modelling of fDLPs

Iterative Threading ASSEmbly Refinement (I-TASSAR) server was employed for *ab initio* modelling (http://zhanglab.ccmb.med.umich.edu/I-TASSER). As an online platform for protein structure and function predictions, I-TASSER was ranked as the No. 1 server for protein structure prediction in recent community-wide CASP7, CASP8, CASP9, CASP10 and CASP11 experiments [14] (http://zhanglab.ccmb.med.umich.edu/I-TASSER). The confidence of each model is quantitatively measured by C-score that was calculated based on the significance of threading template alignments and the convergence parameters of the structure assembly simulations. Swiss-PdbViewer (http://spdbv.vital-it.ch/) was used to connect adjacent unpaired cysteines and to perform final energy minimization.

### Synthesis, oxidative refolding and characterization of Zytrisin-1

Zytrisin-1 was chemically synthesized in its reduced form by ChinaPeptides (Shanghai, China) and oxidative refolding was performed according to the method previously described [7]. Oxidized Zytrisin-1 was purified to homogeneity by reversed phase high pressure liquid chromatography (RP–HPLC). Purity and molecular masses of the peptide were determined by matrix-assisted laser desorption ionization time-of-flight mass spectrometry (MALDI-TOF MS) on a Kratos PC Axima CFR plus (Shimadzu Co. LTD, Kyoto, Japan).

Circular dichroism (CD) spectra of reduced and oxidized Zytrisin-1 were recorded on Chirascan™-plus circular dichroism spectrometer (Applied Photophysics Ltd, U.K.) at room temperature from 190 to 260 nm with a quartz cell of 1.0 mm thickness. Data were collected at 1 nm intervals with a scan rate of 60 nm/min. CD data are expressed as mean residue molar ellipticity (θ).

Antimicrobial activity of oxidized Zytrisin-1 was evaluated by the inhibition zone assay [7]. Microbial strains used here include five Gram-positive bacteria (*Bacillus megaterium, Micrococcus luteus, Bacillus subtilis, Staphylococcus aureus* and *S. aureus* P1386); four Gram-negative bacteria (*Escherichia coli* ATCC 25922, *Pseudomonas aeruginosa, Serratia marcescens* and *Xanthomonas oryzae*) and two fungi (*Neurospora crassa* and *Candida albicans* JX1195).

## Results

**Figure 1:**
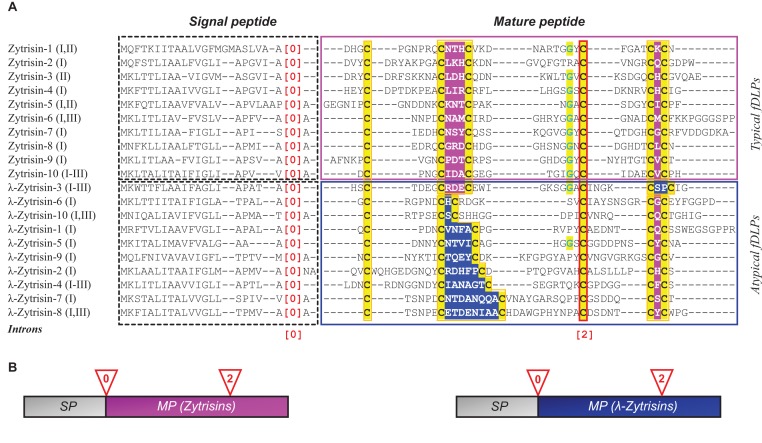
Comparison of amino acid sequences and gene structures of Zytrisins and λ-Zytrisins (**A**) Multiple sequence alignments of representative fDLPs from *Z. tritici*. Signal peptides and mature peptides are indicated by dotted and solid boxes respectively. Cysteines are shadowed in yellow and the glycines at -2 of the fourth cysteine is shown in cyan and shadowed in yellow. The fourth cysteine disrupted by a conserved phase-2 intron is boxed in red. [0] represents phase-0 intron. I, II and III represent isolates of *Z. tritici* – IPO323, STIR04 A48b and STIR04 A26b respectively. Owing to mutations among isolates, some allele genes might be evolutionarily lost, as Zytrisin-1 (I,II) in isolate III, but some retain identical sequences among the three isolates, e.g. Zytrisin-10, λ-Zytrisin-3 and λ-Zytrisin-4. The CSαβ motifs (CXXXC and CXC) in Zytrisins are shadowed in pink and the altered motifs in λ-Zytrisins in blue. (**B**) Schematic diagram of gene structures of Zytrisins and λ-Zytrisins. SP, signal peptide; MP, mature peptide.

**Figure 2:**
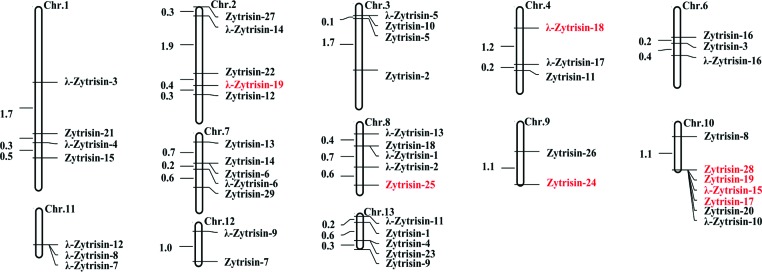
The chromosome map of Zytrisin and λ-Zytrisin from *Z. tritici* IPO323 The numbers on the chromosome indicates physical distance of each gene with one million base pairs as a unit. Predicted pseudogenes are highlighted in red. The map was drawn by Mapdraw [36].

*Zymoseptoria* mainly includes some phytopathogenic fungi. For instance, *Zymoseptoria tritici* is the causal agent of the septoria tritici blotch (STB) in wheat that ranks as one of the most economically important diseases [15,16]. From genomes of three isolates of *Z. tritici* (IPO323, STIR04 A48b and STIR04 A46b) sequenced recently, we identified a total of 29 genes encoding typical fDLPs (named Zytrisins) (see Appendixes 1 and 2 in the Supplementary Data), which hold the conserved cystine framework (Figure 1A). Genomic organization analysis revealed that all the Zytrisin genes possess a conserved exon–intron structure, including a phase-0 intron located at the end of a signal peptide and a phase-2 intron interrupting the codon of the fourth cysteine (Figure 1B). Remarkably, 19 genes encoding peptides with an altered Cys_[2]_X_3_Cys_[3]_ or Cys_[5]_X_1_Cys_[6]_ motif were also identified, which had different protein sequence spaces (residue numbers) between the cysteines of the motifs (Figure 1). We designated these atypical fDLPs λ-Zytrisins, whose variable sequence spaces vary from two to nine amino acids between Cys_[2]_ and Cys_[3]_ and two between Cys_[5]_ and Cys_[6]_. Despite this, they all have an identical exon–intron structure with that of Zytrisins. The precise location and phase conservation in their introns are a key relic of homology between Zytrisins and λ-Zytrisins [17,18], in agreement with their identical precursor organization and six conserved cysteines (Figure 1). In certain isolates, Zytrisin genes became pseudogenes by the loss of the initiation codon or mutation into a premature termination codon (PTC) or insertion/deletion (indel)-mediated frame-shift mutations (e.g. *Zytrisin-2* in STIR04 A48b and STIR04 A46b; *Zytrisin-15* in STIR04 A46b; *Zytrisin-17, -19* and *-28* in IPO323; and *Zytrisin-24* in all the three isolates) (Appendix 1). SRA-Blast of the recently updated RNA-seq data of *Z. tritici* IPO323 [19] confirmed the presence of the two predicted introns and showed that these *Zytrisin* and *λ-Zytrisin* genes all are transcriptionally active with the exception of the pseudogenes listed here (Appendix 1). In addition, we found that some *Zytrisin* and *λ-Zytrisin* genes are arranged adjacently on chromosomes (Figure 2), further supporting their evolutionary relationship, possibly originated via gene duplication followed by sequence modification.

**Figure 3:**
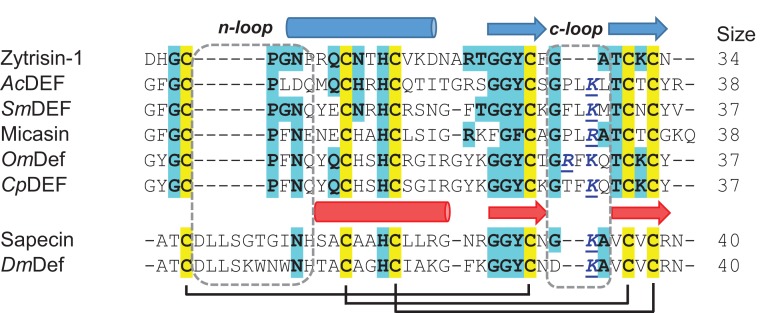
Multiple sequence alignment of Zytrisin-1 and other known defensins AITDs: *Ac*DEF (P80154); *Sm*DEF (KFM61041), micasin (AEM44801), *Cp*DEF (ACJ04429), *Om*Def (BAB41027); CITDs: sapecin (AAA29984); *Dm*Def (AAO72492). Conserved cysteines are shadowed in yellow and identical residues to Zytrisin-1 in cyan. Secondary structure elements and disulfide bridges are extracted from the structural coordinates of Micasin (PDB ID: 2LR5) and Sapecin (PDB ID: 1L4V). Two loops are boxed in dotted lines. The functionally important basic residues in the c-loop are coloured in blue.

**Figure 4:**
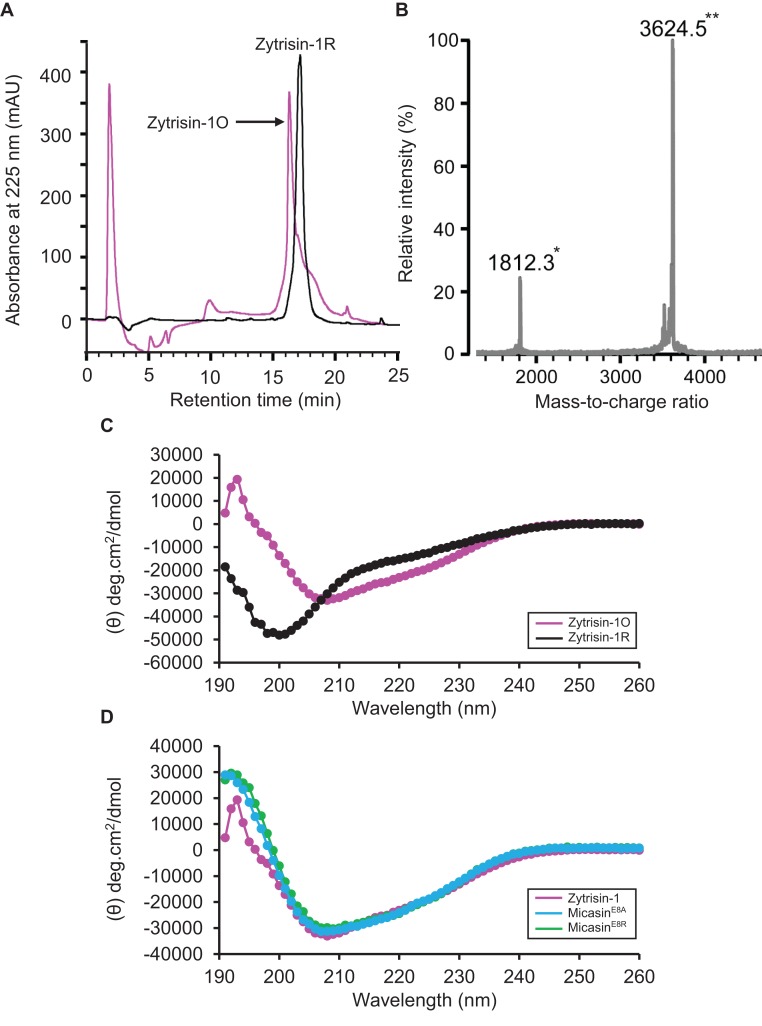
Characterization of synthetic Zytrisin-1 (**A**) RP–HPLC showing retention time of reduced and oxidized Zytrisin-1, named Zytrisin-1R and Zytrisin-1O respectively. (**B**) Determination of the MW of oxidized Zytrisin-1 by MALDI-TOF MS. The spectra had two main peaks, corresponding to the singly (*) and doubly (**) protonated forms of the peptide. (**C**) Comparison of the CD spectra between reduced and oxidized Zytrisin-1. (**D**) Comparison of the CD spectra of oxidized Zytrisin-1 with those of Micasin^E8A^ and Micasin^E8R^ [21]. The spectra were measured at a peptide concentration of 0.05–0.1 mg/ml in water.

Sequence analysis revealed that with the exception of the six conserved cysteines, other sites exhibited high variability between Zytrisins and λ-Zytrisins, even within the same group (Figure 1). Further database search indicated that they both lacked significant sequence similarity to proteins deposited in GenBank (http://www.ncbi.nlm.nih.gov/) and SWISS-PROT (http://www.expasy.org/) except Zytrisin-1 that shows detectable sequence similarity and identical n-loop size to AITDs [12] (Figure 3). Interestingly, its c-loop is closer to CITDs than to AITDs (Figure 3), suggesting its evolutionary position between these two types of defensins [20]. To study the structural and functional features of this peptide, we prepared highly pure native-like Zytrisin-1 through chemical synthesis and oxidative refolding (Figure 4A). This peptide in its reduced form had an experimental molecular weight (MW) of 3629.10 Da determined by ESI-MS, in line with its theoretical MW of 3629.03 Da. Its oxidized product had an experimental MW of 3624.5 Da determined by MALDI-TOF (Figure 4B), perfectly matching its theoretical MW of 3623.0 Da. The native-like structure of this peptide was verified by CD analysis. As shown in Figure 4(C), the reduced peptide remained a random coli conformation because its CD spectra had a minimum at 200 nm. However, the CD spectra of its oxidized product significantly changed, as identified by a minimum at 208 nm and a maximum at 193 nm, indicative of a typical CSαβ fold [7]. This is further strengthened by its similarity to the CD spectra of Micasin^E8A^ and Micasin^E8R^ (Figure 4D), two structurally and functionally known mutants of micasin from the dermatophytic fungus *Microsporum canis* [22]. Inhibition zone assay indicated that Zytrisin-1 lacked inhibitory activity on a series of bacterial and fungal strains used here (see Materials and methods section) at a dose of 0.4–0.8 nmol each well. The functional loss could be related to its c-loop, a region located within the functional γ-core of AITDs and CITDs [22], where a cationic residue is a premise for the function of these defensins [23]. Obviously, this peptide lacks such a residue (Figure 3). Thus, its biological function beyond antibacterial immunity remains to be established in the future.

Homologues of Zytrisins and λ-Zytrisins were also identified in three sister species of *Z. tritici* (*Z. pseudotritici*; *Z. ardabiliae* and *Z. passerinii*). Numbers of these fDLPs in each species are listed in Table 1 and their locations in genomes are collected in Appendix 1, and all protein sequences are provided in Appendix 2–5, as supplementary information. It is worth mentioning that in *Z. ardabiliae* there are fDLPs containing two defensin domains, named Bizyarsin (Supplementary Figure S1). In total, we mined 195 fDLP genes, including some pseudogenes in *Zymoseptoria* (Appendix 1 in the Supplementary Data). Among them, more than 70% of members carry net negative or neutral charges under pH 7.0 (Table 1), which are different from defensins with antibacterial activity [7]. These defensins are usually cationic and need positively charged residues to interact with anionic bacterial membrane [24].

**Table 1:** Gene numbers of fDLPs in different species of *Zymoseptoria*

	Isolates	Typical fDLPs		Atypical fDLPs		Total number
		Name	Number	Name	Number	
*Z. tritici*	3	Zytrisin	29	λ-Zytrisin	19	48 (73%)
*Z. pseudotritici*	5	Zypsesin	28	λ-Zypsesin	15	43 (76%)
*Z. ardabiliae*	4	Zyarsin	50	λ-Zyarsin	20	70 (90%)
*Z. passerinii*	1	Zypassin	25	λ-Zypassin	9	34 (75%)

Note: Percentages in brackets indicate ratios of positively charged peptides in each group.

**Figure 5:**
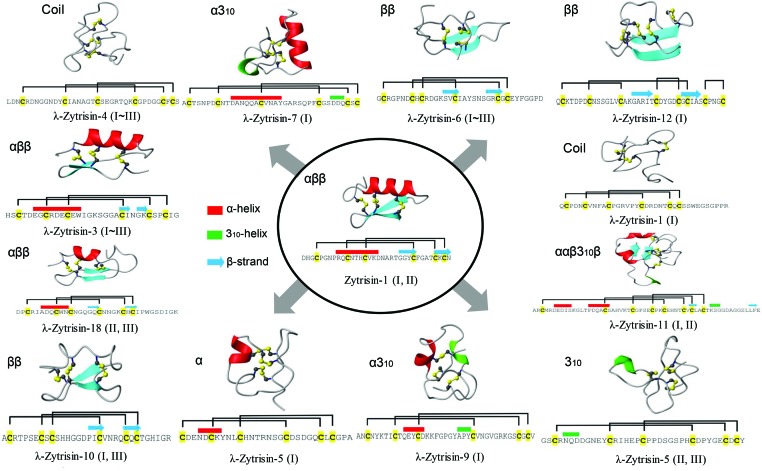
Computational model structures of Zytrisins and λ-Zytrisins Cysteines in sequences are shadowed in yellow. Secondary structures (cylinder: α-helix; arrow: β-strand) and disulfide bridges are extracted from the modelled structures. Fold types for each peptide are presented on the upper left of their structures. 3_10_, a type of α-helical structure found in proteins and polypeptides, which contains three residues per turn and ten atoms between hydrogen bond donor and acceptor [37].

From a structural viewpoint, the formation of the CSαβ fold depends on the motifs (CysX_3_Cys/CysX_1_Cys) to provide precise disulfide bridge locations for stabilizing the structure [24]. Given this fact, we anticipated that λ-Zytrisins might have changed their folds. To confirm this assumption, we built structural models of these peptides and chose Zytrisin-1 as a representative of typical fDLPs. Considering the absence of suitable structural templates for λ-Zytrisins due to low sequence similarity, we employed an *ab initio* protein structure modelling method on the server of I-TASSER to obtain models of all the peptides, including Zytrisin-1. The C-scores of these successfully modelled structures range from −2.5 to −0.09 [14]. Because C-score is typically in the range of (−5, 2) for the I-TASSER models, our computational structures were assumed reliable (Figure 5). This is also confirmed by the model of Zytrisin-1 that shows a typical defensin structure with three identical disulfide bridges (Figure 5) and is highly similar to the experimental structure of micasin, in line with their CD data (Figure 4D).

Analysis of these structures led to the identification of three different disulfide connectivity patterns in λ-Zytrisins, including: (1) Cys_[1]_–Cys_[5]_; Cys_[2]_–Cys_[4]_; Cys_[3]_–Cys_[6]_ in λ-Zytrisin-4, -6 and -7. This pattern is identical with vertebrate β-defensins [3]; (2) Cys_[1]_–Cys_[4]_; Cys_[2]_–Cys_[5]_; Cys_[3]_–Cys_[6]_ for most λ-Zytrisins. This pattern is identical with Zytrisin-1 and other typical fDLPs; (3) Cys_[1]_–Cys_[3]_; Cys_[2]_–Cys_[6]_; Cys_[4]_–Cys_[5]_ in λ-Zytrisin-12 (Figure 5). The change in the disulfide pattern was also proposed in the evolution of a nematode-derived CSαβ defensin (ASABF-α) [25]. However, this change, if any, is achieved by deleting cysteines other than indels of other types of amino acids in a conserved cystine framework (such as λ-Zytrisins). In addition, snake venom-derived disintegrins functioning as antagonists of platelet aggregation, have a non-canonical disulfide bridge pattern among different groups (i.e. albolabrin/saxatilin/salmosin). However, it appears that the presence of this disulfide bridge leads to no fold change [26, 27]. Conversely, λ-Zytrisins exhibit a diversity of fold types due to the motif changes, ranging from αββ to ββ, α, 3_10_, α3_10_, ααβ3_10_β and coil (Figure 5). It is noteworthy that although the fold in λ-Zytrisin-3 and λ-Zytrisin-18 is still arranged as αββ and their disulfide bridges are also identical with typical fDLPs, positions of their secondary structure elements significantly changes (Figure 5). The fold change in λ-Zytrisins is also consistent with a glycine mutation at -2 position relative to the fourth cysteine (GlyXCys_[4]_) (Figure 1). In the majority of CSαβ peptides, this position is dominated by a glycine due to space limitation to only one hydrogen atom accommodated at intersection of the α-helix and β-strand (see Figure 5) [28] and thus introducing a larger side chain will lead to structural change to remove its steric hindrance.

**Figure 6:**
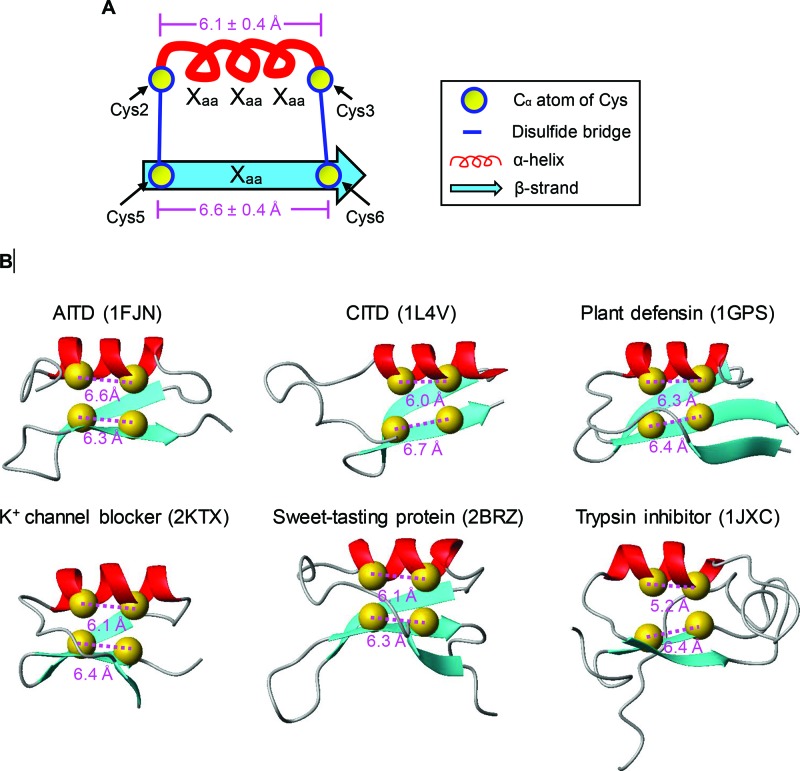
Distances between Cα atoms of cysteines in the CysX_3_Cys or CysX_1_Cys motifs (**A**) Diagram representation illustrating the distances of cysteines spanning the α-helix and the β-strand, expressed as mean ± S.D. (**B**) Representative CSαβ structures with the distances shown.

To provide a mechanical explanation for the fold change in the atypical defensins, we analysed experimental structures of 48 CSαβ peptides (two determined by X-ray crystallography and 46 by NMR) to calculate the distance between two cysteine C_α_ atoms in the two motifs (CysX_3_Cys/CysX_1_Cys). These CSαβ peptides cover a wide range of functional classes, including scorpion toxins affecting K^+^, Na^+^ and Cl^−^ channels, defensins from plants, fungi and invertebrates and the sweet-tasting protein and trypsin inhibitor from plants [10]. Their sequences are provided in Supplementary Figure S2. As shown in Figure 6, the distance between the two C_α_ atoms of CysX_3_Cys of the peptides is highly similar, with a value of 6.1 ± 0.4 Å, whereas the corresponding value in the CysX_1_Cys motif is 6.6 ± 0.4 Å (Figure 6). This observation indicates that the formation of disulfide bridges between the curving α-helix spanning CysX_3_Cys and the extended β-strand striding CysX_1_Cys are constrained by suitable distances. Insertion or deletion of residues in spacing of two cysteines in these motifs certainly will add or reduce the distances. These would lead to a difficulty in forming the initial disulfide bridges if the α-helix and β-strands were not changed. Apart from the alteration in secondary structure elements, some members also display a different disulfide bridge pattern due to the distance change (Figure 5).

## Discussion

The present study might challenge the prevailing view that the evolution of peptides mainly involves indels in loops without alterations in their structural cores [29]. The atypical fDLPs described here not only extensively modify their loops, but also alter their core elements (Figures 1 and 5). Apart from the small peptides, it was also proposed that some folds might change along the evolution of homologous proteins [30]. However, in these examples the investigated proteins had rather low sequence similarity hampering the establishment of reliable homology in the absence of other evidence. Our finding that Zytrisins and λ-Zytrisins have explicit homology signals but fold differently provides a clear example for divergent evolution of fDLPs at the fold level, and it will be helpful in establishing evolutionary relationship among different types of defensins from both vertebrates and invertebrates. For example, α- and β-defensins are two important innate immunity components of vertebrates, but their evolutionary relationship remains unsolved due to the differences in the disulfide bridge pattern and fold type though their homology is widely supported by genomic position, function and phyletic distribution [31, 32, 33]. With our observation presented here, structural differences between these two types of defensins are no longer a barrier for elucidating their paralogous relationship.

In a recent study, Shafee et al. [2] proposed the concept of *cis-* and *trans*-defensins and suggested they represent two independent and convergent protein superfamilies due to the difference in the pattern of disulfide connectivities. However, the results of our present study clearly demonstrate that this pattern and even the fold type are changeable in evolution of defensins. Hence, the possibility of divergence of all the defensins from a common ancestor is not ruled out currently. Given that big defensins, the only one invertebrate-derived member in *trans*-defensins, have been considered as the ancestor of vertebrate β-defensins [3], we propose that this class of immune peptides could be an evolutionary intermediate linking the *cis*- and *trans*-defensins. A possible evolutionary trajectory can be thus elucidated as follows: a CSαβ-type defensin ancestor firstly developed into a big defensin in invertebrates and then β-defensins appeared following the emergence of vertebrates [3] and evolved to α-defensins by gene duplication and mutations, which finally evolved to θ-defensins in primates [34]. The presence of β-defensin-like disulfide bridges in certain λ-Zytrisins (Figure 5) provides an example of disulfide pattern switch among different defensin classes. However, to draw a more decisive conclusion, experimental conversion between *cis-* and *trans*-defensins will be definitely necessary.

It is known that the CSαβ-type of defensins from plants exhibit a variety of biological activities [35]. In addition to the capacity in eliminating microbial infections, some members are found to act as enzyme inhibitors and zinc tolerance mediators, and even regulators of plant growth and development [35]. In fungi, at least two defensins (pechrysin and anisin-1) have lost their antibacterial activity and the *anisin-1* gene is clearly associated with the fitness of *Aspergillus nidulans* [8,9]. In terms of *Zymoseptoria* fDLPs, their overall anionic feature might rule out their roles in immune response of this genus of fungi. Regardless the biological activity, the discovery of structural change in evolution of fDLPs will enhance our understanding of peptide evolution and be valuable in guiding design of new folds through rational adjustment of structural core of a protein.

## Supplementary information

Supplementary information includes two figures (Supplementary Figure S1 and Supplementary Figure S2), one table (Supplemenatry Table S1) and five appendix files (Appendixes 1–5). All structural models built here will be provided upon request.

## Abbreviations

AITD: ancient invertebrate-type defensin
ASABF: *Ascaris suum* antibacterial factor
CASP: critical assessment of protein structure prediction
CD: circular dichroism
CITD: classical insect-type defensin
CSαβ: cystine-stabilized α-helix and β-sheet
ESI-MS: electrospray ionization mass spectrometry
fDLP: fungal defensin-like peptide
I-TASSER: Iterative Threading ASSEmbly Refinement
MALDI-TOF MS: matrix-assisted laser desorption ionization time-of-flight mass spectrometry
MW: molecular weight
PTC: premature termination codon
RP–HPLC: reversed phase-high pressure liquid chromatography
SRA: sequence read archive

## References

[1] Grishin D.V. and Sokolov N.N. (2014) Defensins – natural peptide antibiotics of higher eukaryotes. Biomed. Khim. 60, 438–447 25249527 10.18097/pbmc20146004438

[2] Shafee T.M., Lay F.T., Hulett M.D. and Anderson M.A. (2016) The defensins consist of two independent, convergent protein superfamilies. Mol. Biol. Evol. 33, 2345–2356 27297472 10.1093/molbev/msw106

[3] Zhu S. and Gao B. (2013) Evolutionary origin of β-defensins. Dev. Comp. Immunol. 39, 79–84 22369779 10.1016/j.dci.2012.02.011

[4] Galagan J.E., Henn M.R., Ma L.J., Cuomo C.A. and Birren B. (2005) Genomics of the fungal kingdom: insights into eukaryotic biology. Genome Res. 15, 1620–1631 16339359 10.1101/gr.3767105

[5] Mygind P.H., Fischer R.L., Schnorr K.M., Hansen M.T., Sönksen C.P., Ludvigsen S. (2005) Plectasin is a peptide antibiotic with therapeutic potential from a saprophytic fungus. Nature 437, 975–980 16222292 10.1038/nature04051

[6] Oeemig J.S., Lynggaard C., Knudsen D.H., Hansen F.T., Nørgaard K.D., Schneider T. (2012) Eurocin, a new fungal defensin: structure, lipid binding, and its mode of action. J. Biol. Chem. 287, 42361–42372 23093408 10.1074/jbc.M112.382028 PMC3516779

[7] Zhu S., Gao B., Harvey P.J. and Craik D.J. (2012) Dermatophytic defensin with antiinfective potential. Proc. Natl. Acad. Sci. U.S.A. 109, 8495–8500 22586077 10.1073/pnas.1201263109 PMC3365176

[8] Eigentler A., Pocsi I. and Marx F. (2012) The anisin1 gene encodes a defensin-like protein and supports the fitness of *Aspergillus nidulans* . Arch. Microbiol. 194, 427–437 22113351 10.1007/s00203-011-0773-y PMC3354322

[9] Wu Y., Gao B. and Zhu S. (2014) Fungal defensins, an emerging source of anti-infective drugs. Chinese Sci. Bull. 59, 931–935

[10] Zhu S., Gao B. and Tytgat J. (2005) Phylogenetic distribution, functional epitopes and evolution of the CSαβ superfamily. Cell. Mol. Life Sci. 62, 2257–2269 16143827 10.1007/s00018-005-5200-6 PMC11138386

[11] Zhu S., Peigneur S., Gao B., Umetsu Y., Ohki S. and Tytgat J. (2014) Experimental conversion of a defensin into a neurotoxin: implications for origin of toxic function. Mol. Biol. Evol. 31, 546–559 24425781 10.1093/molbev/msu038

[12] Zhu S. (2008) Discovery of six families of fungal defensin-like peptides provides insights into origin and evolution of the CSαβ defensins. Mol. Immunol. 45, 828–838 17675235 10.1016/j.molimm.2007.06.354

[13] Wu J., Gao B. and Zhu S. (2014) The fungal defensin family enlarged. Pharmaceuticals (Basel) 7, 866–880 25230677 10.3390/ph7080866 PMC4165938

[14] Roy A., Kucukural A. and Zhang Y. (2010) I-TASSER: a unified platform for automated protein structure and function prediction. Nat. Protoc. 5, 725–738 20360767 10.1038/nprot.2010.5 PMC2849174

[15] Goodwin S.B., M’Barek S.B., Dhillon B., Wittenberg A.H., Crane C.F., Hane J.K. (2011) Finished genome of the fungal wheat pathogen *Mycosphaerella graminicola* reveals dispensome structure, chromosome plasticity, and stealth pathogenesis. PLoS Genet. 7, e1002070 21695235 10.1371/journal.pgen.1002070 PMC3111534

[16] Quaedvlieg W., Kema G.H., Groenewald J.Z., Verkley G.J., Seifbarghi S., Razavi M. et al. (2011) *Zymoseptoria* gen. nov.: a new genus to accommodate Septoria-like species occurring on graminicolous hosts. Persoonia 26, 57–69 22025804 10.3767/003158511X571841 PMC3160802

[17] Betts M.J., Guigo R., Agarwal P. and Russell R.B. (2001) Exon structure conservation despite low sequence similarity: a relic of dramatic events in evolution? EMBO J. 20, 5354–5360 11574467 10.1093/emboj/20.19.5354 PMC125659

[18] Rogozin I.B., Sverdlov A.V., Babenko V.N. and Koonin E.V. (2005) Analysis of evolution of exon-intron structure of eukaryotic genes. Brief Bioinform. 6, 118–134 15975222 10.1093/bib/6.2.118

[19] Kellner R., Bhattacharyya A., Poppe S., Hsu T.Y., Brem R.B. and Stukenbrock E.H. (2014) Expression profiling of the wheat pathogen *Zymoseptoria tritici* reveals genomic patterns of transcription and host-specific regulatory programs. Genome Biol. Evol. 6, 1353–1365 24920004 10.1093/gbe/evu101 PMC4079195

[20] Gao B. and Zhu S. (2012) Alteration of the mode of antibacterial action of a defensin by the amino-terminal loop substitution. Biochem. Biophys. Res. Commun. 426, 630–635 22975352 10.1016/j.bbrc.2012.08.143

[21] Wu J., Gao B. and Zhu S. (2016) Single-point mutation-mediated local amphipathic adjustment dramatically enhances antibacterial activity of a fungal defensin. FASEB J. 30, 2602–2614 27084888 10.1096/fj.201500157

[22] Yount N.Y. and Yeaman M.R. (2004) Multidimensional signatures in antimicrobial peptides. Proc. Natl. Acad. Sci. U.S.A. 101, 7363–7368 15118082 10.1073/pnas.0401567101 PMC409924

[23] Romestand B., Molina F., Richard V., Roch P. and Granier C. (2003) Key role of the loop connecting the two β-strands of mussel defensin in its antimicrobial activity. Eur. J. Biochem. 270, 2805–2813 12823551 10.1046/j.1432-1033.2003.03657.x

[24] Tamaoki H., Miura R., Kusunoki M., Kyogoku Y., Kobayashi Y. and Moroder L. (1998) Folding motifs induced and stabilized by distinct cystine frameworks. Protein Eng. 11, 649–659 9749917 10.1093/protein/11.8.649

[25] Minaba M., Ueno S., Pillai A. and Kato Y. (2009) Evolution of ASABF (*Ascaris suum* antibacterial factor)-type antimicrobial peptides in nematodes: putative rearrangement of disulfide bonding patterns. Dev. Comp. Immunol. 33, 1147–1150 19560487 10.1016/j.dci.2009.06.011

[26] Hong S.Y., Sohn Y.D., Chung K.H. and Kim D.S. (2002) Structural and functional significance of disulfide bonds in saxatilin, a 7.7 kDa disintegrin. Biochem. Biophys. Res. Commun. 293, 530–536 12054633 10.1016/S0006-291X(02)00258-9

[27] Calvete J.J., Marcinkiewicz C., Monleon D., Esteve V., Celda B. and Juarez P. (2005) Snake venom disintegrins: evolution of structure and function. Toxicon 45, 1063–1074 15922775 10.1016/j.toxicon.2005.02.024

[28] Bontems F., Roumestand C., Gilquin B., Menez A. and Toma F. (1991) Refined structure of charybdotoxin: common motifs in scorpion toxins and insect defensins. Science 254, 1521–1523 1720574 10.1126/science.1720574

[29] Menez A., Bontems F., Roumestand C., Gilquin B. and Toma F. (1992) Structural basis for functional diversity of animal toxins. Proc. Roy. Soc. Edinb. B 99, 83–103

[30] Grishin N.V. (2001) Fold change in evolution of protein structures. J. Struct. Biol. 134, 167–185 11551177 10.1006/jsbi.2001.4335

[31] Liu L., Zhao C., Heng H.H. and Ganz T. (1997) The human β-defensin-1 and α-defensins are encoded by adjacent genes: two peptide families with differing disulfide topology share a common ancestry. Genomics 43, 316–320 9268634 10.1006/geno.1997.4801

[32] Hughes A.L. (1999) Evolutionary diversification of the mammalian defensins. Cell. Mol. Life Sci. 56, 94–103 11213266 10.1007/s000180050010 PMC11147084

[33] Xiao Y., Hughes A.L., Ando J., Matsuda Y., Cheng J.F. and Skinner-Noble D. (2004) A genome-wide screen identifies a single β-defensin gene cluster in the chicken: implications for the origin and evolution of mammalian defensins. BMC Genomics 5, 56 15310403 10.1186/1471-2164-5-56 PMC515299

[34] Li D., Zhang L., Yin H., Xu H., Satkoski Trask J., Smith D.G. et al. (2014) Evolution of primate α and θ defensins revealed by analysis of genomes. Mol. Biol. Rep. 41, 3859–3866 24557891 10.1007/s11033-014-3253-z

[35] Carvalho Ade O. and Gomes V.M. (2009) Plant defensins-prospects for the biological functions and biotechnological properties. Peptides 30, 1007–1020 19428780 10.1016/j.peptides.2009.01.018

[36] Liu R.H. and Meng J.L. (2003) MapDraw: a microsoft excel macro for drawing genetic linkage maps based on given genetic linkage data. Yi Chuan 25, 317–321 15639879

[37] Karpen M.E., De Haseth P.L. and Neet K.E. (1992) Differences in the amino acid distributions of 3_10_-helices and α-helices. Protein Sci. 1, 1333–1342 1303752 10.1002/pro.5560011013 PMC2142095

